# Focused ultrasound: an effective technique for unleashing the power of immunotherapy in the tumor microenvironment?

**DOI:** 10.1186/2050-5736-3-S1-O39

**Published:** 2015-06-30

**Authors:** Elizabeth Repasky

**Affiliations:** 1 Roswell Park Cancer Institute, Buffalo, NY, United States

## Background/introduction

The tumor microenvironment presents significant barriers to the infiltration of anti-tumor immune effector cells as well as to the delivery of cancer therapeutics. In part, physical parameters such as high interstitial pressure, defective vascular elements and abnormally dense stromal elements contribute significantly to poor uptake of immune cells. However, growing evidence also shows that tumor-cells can produce inflammatory cytokines as well as express certain surface receptors that drive both the accumulation of immunosuppressive cells as well as block the activation of antigen-specific immune effector cells, such as CD8+ T cells, which are able to traverse the physical barriers to the interior of tumors.

## Methods

In a brief introduction to this session, I will highlight some exciting new advances in tumor immunology/immunotherapy as well as outline several properties of focused ultrasound (FUS) that could make it a very attractive immune adjuvant that can non-invasively manipulate the tumor microenvironment for enhanced anti-tumor immunity. I will also highlight several recent publications which strongly support the ability of FUS to stimulate endogenous anti-tumor immunity and support its combination with immunotherapy.

